# Possible Spreading of SARS-CoV-2 from Humans to Captive Non-Human Primates in the Peruvian Amazon

**DOI:** 10.3390/ani14050732

**Published:** 2024-02-27

**Authors:** Andrea Tavera Gonzales, Jhonathan Bazalar Gonzales, Thalía Silvestre Espejo, Milagros Leiva Galarza, Carmen Rodríguez Cueva, Dennis Carhuaricra Huamán, Luis Luna Espinoza, Abelardo Maturrano Hernández

**Affiliations:** 1 Research Group in Biotechnology Applied to Animal Health, Production and Conservation (SANIGEN), Laboratorio de Biología y Genética Molecular, Facultad de Medicina Veterinaria, Universidad Nacional Mayor de San Marcos, Lima 15021, Peru; 2 Asociación Equipo Primatológico del Perú, Iquitos 16008, Peru; 3 Programa de Pós-Graduação Interunidades em Bioinformática, Instituto de Matemática e Estatística, Universidade de São Paulo, Rua do Matão 1010, São Paulo 05508-090, Brazil

**Keywords:** SARS-CoV-2, metagenomics, non-human primates, Peruvian Amazon

## Abstract

**Simple Summary:**

Numerous instances of SARS-CoV-2 transmission from humans to both domestic and wild animals have been reported globally. However, in Peru, natural infections in wild animals have not been documented, despite the high number of COVID-19 cases and deaths in humans, particularly in the Amazonian regions where interactions between humans and wildlife are frequent. In this study, we conducted SARS-CoV-2 surveillance using fecal samples from 76 captive and semi-captive monkeys in the Loreto, Ucayali, and Madre de Dios regions from August 2022 to February 2023. We identified a genetic segment of SARS-CoV-2 through metagenomic sequencing in a pooled fecal sample from captive white-fronted capuchins (*Cebus unicolor*) at a rescue center in Ucayali. Phylogenetic analysis further confirmed that the obtained sequence matched the SARS-CoV-2 genome. This study represents the initial report of molecular detection of SARS-CoV-2 in monkeys in the Peruvian Amazon, underscoring the adverse impact of human–wildlife interactions and emphasizing the importance of continuous surveillance for the early detection and anticipation of potential future emergencies involving new variants of SARS-CoV-2 in wildlife.

**Abstract:**

Human-to-animal transmission events of SARS-CoV-2 (Severe Acute Respiratory Syndrome Coronavirus 2) have been reported in both domestic and wild species worldwide. Despite the high rates of contagion and mortality during the COVID-19 (Coronavirus Diseases 2019) pandemic in Peru, no instances of natural virus infection have been documented in wild animals, particularly in the Amazonian regions where human–wildlife interactions are prevalent. In this study, we conducted a surveillance investigation using viral RNA sequencing of fecal samples collected from 76 captive and semi-captive non-human primates (NHPs) in the Loreto, Ucayali, and Madre de Dios regions between August 2022 and February 2023. We detected a segment of the RNA-dependent RNA polymerase (RdRp) gene of SARS-CoV-2 by metagenomic sequencing in a pooled fecal sample from captive white-fronted capuchins (*Cebus unicolor*) at a rescue center in Bello Horizonte, Ucayali. Phylogenetic analysis further confirmed that the retrieved partial sequence of the RdRp gene matched the SARS-CoV-2 genome. This study represents the first documented instance of molecular SARS-CoV-2 detection in NHPs in the Peruvian Amazon, underscoring the adverse impact of anthropic activities on the human–NHP interface and emphasizing the importance of ongoing surveillance for early detection and prediction of future emergence of new SARS-CoV-2 variants in animals.

## 1. Introduction

The severe acute respiratory syndrome coronavirus 2 (SARS-CoV-2) emerged in Wuhan at the end of 2019 and rapidly disseminated worldwide, leading to the COVID-19 pandemic [1]. Peru was among the nations most severely affected, experiencing high rates of contagion and mortality due to the early spread of the virus and community transmission [2,3]. As of July 2023, the total number of confirmed deaths in the country had reached 221,368. Among these, 8709 occurred in the Loreto, Ucayali, and Madre de Dios regions, situated in the Peruvian Amazon, accounting for approximately 5% of the Peruvian population [3]. The COVID-19 pandemic had a profound and devastating impact on the population of the Peruvian Amazon, an area known for its rich biodiversity and wildlife [4].

Numerous studies have demonstrated the transmission of SARS-CoV-2 from humans to domestic animals such as dogs, cats, ferrets, and hamsters [5,6,7]. Furthermore, SARS-CoV-2 has been identified in zoo animals, including lions, tigers, wild minks, otters, and great apes [8,9,10,11]. There have even been documented cases of extended transmission among farmed minks in Europe and white-tailed deer in North America [12,13]. Despite reduced human contact, the virus has also been detected in wild white rhinoceros in Africa [14]. The introduction of SARS-CoV-2 into animal hosts is of great concern because it can lead to the accumulation of new mutations, and this process has the potential to modify its pathogenicity, virulence, and its ability to evade pre-existing immune responses in humans. Consequently, this may lead to the reintroduction of viruses into susceptible human populations [15]. However, there is still limited evidence to confirm the emergence of a variant of SARS-CoV-2 of interest or concern from animal hosts, although we cannot rule out the possibility that this could occur in the future, emphasizing the importance of ongoing monitoring of the virus in new animal species [16].

In non-human primates (NHP), experimental infections in Rhesus macaques (*Macaca mulatta*) and Cynomolgus macaques (*Macaca fascicularis*) have demonstrated the development of characteristic COVID-19 lesions, identified through computed tomography scans, even in the absence of clinical signs [17]. Additionally, a natural infection by SARS-CoV-2 was reported in a Black-Tailed Marmoset (*Mico melanurus*) from Brazil, suggesting that NHPs could potentially serve as reservoirs and vectors for SARS-CoV-2 [18]. These instances underscore the importance of conducting virus surveillance within NHP populations to identify potential reservoirs and evaluate the potential risk of zoonotic transmission [19].

In Peru, there are approximately 42 species of NHPs [7]; however, no reported infections of SARS-CoV-2 or other coronaviruses with zoonotic potential have been documented in these primates. Nevertheless, the possibility of such infections should be considered, given the widespread transmission of SARS-CoV-2 in the Peruvian population. Therefore, between August 2022 and February 2023, we collected fecal samples from captive and semi-captive NHPs in the Loreto, Ucayali, and Madre de Dios regions. Subsequently, we conducted coronavirus molecular detection and metagenomics sequencing of viral RNA to evaluate the potential presence of SARS-CoV-2 as well as other coronaviruses as part of a surveillance study focusing on potential zoonotic pathogens in wildlife.

## 2. Materials and Methods

### 2.1. Sampling Collection

**Figure 1:**
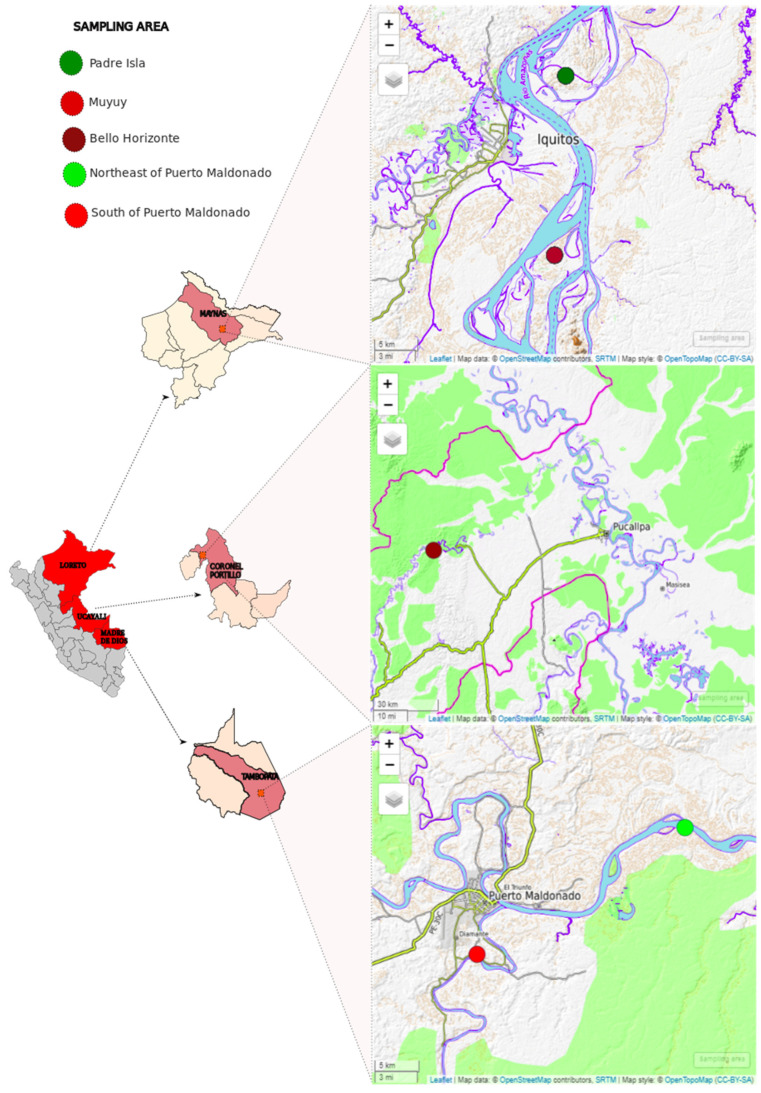
Sampling areas of captive and semi-captive NHPs from islands and rescue centers from Loreto, Ucayali, and Madre de Dios regions, in the Peruvian Amazon rainforest.

Between August 2022 and February 2023, we collected a total of 76 fecal samples from captive and semi-captive, apparently healthy, NHPs in three regions of the Peruvian Amazon: (1) Padre Isla and Muyuy islands in Iquitos-Loreto (semi-captivity), (2) one rescue center in Bello Horizonte-Ucayali (captivity), and (3) two rescue centers in Puerto Maldonado-Madre de Dios (captivity) (Figure 1 and Table 1). Stool samples were obtained by collecting 5 to 10 g of recently excreted stool with a sterile disposable palette. The collection excluded the portion in contact with the floor, and the samples were placed in individual sterile bottles with RNAlater reagent (Thermo Scientific, Waltham, MA, USA) labeled with a sample code. All samples were stored in transport coolers with refrigerating gels and later transferred to the Biology and Molecular Genetics Laboratory of the Veterinary Medicine Faculty at the Universidad Nacional Mayor de San Marcos, where they were stored at −20 °C. Subsequently, the samples were organized into pools according to species and sampling areas (Table 1) and prepared for molecular analysis.

### 2.2. Viral Enrichment and RNA Extraction

Viral enrichment was carried out following the methodology described by [20] with some modifications. Briefly, stool samples were diluted in phosphate-buffered saline (PBS) (1 g in 2000 μL) and vortexed for 10 min until completely mixed; then, they were centrifuged at 14,000× *g* for 10 min at 4 °C. The supernatants were pooled following Table 1. Then, 500 μL of each pool was filtered through a 0.22 µm syringe column filter (Millipore, Burlington, MA, USA). The flow-through was treated with Micrococcal nuclease (2 × 10^6^ gel units/mL, New England Biolabs, Ipswich, MA, USA) and Ribonuclease A (30 mg/mL; Sigma-Aldrich, St. Louis, MO, USA). The mixture reaction was incubated at 45 °C for 15 min and for 1 h at 37 °C, and was immediately followed by extraction using the Quick-RNA Zymo kit (Zymo research, Irvine, CA, USA).

### 2.3. Complementary DNA (cDNA) Synthesis and PCR for Coronavirus Detection

cDNA synthesis was carried out using SupertScript™ IV First-Strand Synthesis System kit (Thermo Fisher Scientific, USA) with random hexamers following the instructions recommended by the manufacturer. Subsequently, 1 U of DNA Polymerase I, Large (Klenow) Fragment, was added, and the reaction was incubated for 15 min at 25 °C, 1 h at 37 °C, and 1 min at 95 °C. cDNA was then subjected to a nested PCR reaction using pan-coronavirus primers targeting the RdRp (*orf1ab* gene) [21]. For the first round of PCR, 5 μL of cDNA was added to a mix reaction containing outer pan coronavirus primers (0.2 μM), 1X PCR Buffer, dNTPs (0.2 mM), Dream *Taq* polymerase (1 U), and nuclease-free water until a final volume of 25 μL. Then, 1 µL of the PCR product was added to a mixture containing internal primers and the same concentrations of reagents used in the previous reaction. First and second rounds of PCR were performed at the following temperatures: 94 °C for 5 min followed by 40 cycles consisting of 94 °C for 40 s, 48 °C for 1 min, and 72 °C for 1 min. The reaction concluded with a final extension step at 72 °C for 10 min. Positive and negative controls (Canine CoV NL-18 vaccine strain and nuclease-free water, respectively) were included. Nested PCR products were analyzed in 2% agarose gel stained with SYBR^TM^ Safe DNA Gel Stain (Invitrogen, Eugene, OR, USA).

### 2.4. Metagenomic Sequencing and Bioinformatic Analysis

Paired-end libraries were prepared from the cDNA obtained from the fecal pools using the Nextera XT Library preparation kit, and sequencing was performed on the MiSeq platform (Illumina, San Diego, CA, USA) with 600 cycles per sequencing read pair (2 × 250 bp).

Poor quality sequences were trimmed using Trimmomatic [22] with LEADING:3 TRAILING:3 SLIDINGWINDOW:4:20 MINLEN:50 as parameters. The sequence data’s quality was checked using FastQC v0.11.5; (http://www.bioinformatics.babraham.ac.uk/projects/fastqc/; accessed on 12 July 2023). Megahit v 1.2.9 [23] was used for de novo assembly of the trimmed sequence data. To identify coronavirus sequences, a BLASTX search was performed using Diamond v 0.9.24 [24] against the coronavirus protein database of RefSeq [25]. To assess that the matched sequence is not a chimerical product of assembly, we mapped the reads against the SARS-CoV-2 Wuhan-1 reference genome (MN908947.3) with Bowtie2 v 2.5.1 [26].

Homologous sequences to those identified in the analysis were retrieved from the NCBI database [27] and utilized for conducting phylogenetic analysis. Multiple alignments were generated using the Aliview program [28]. A phylogenetic tree was constructed using the maximum-likelihood method with a GTR + F + I + G4 nucleotide-substitution model and 1000 bootstrap replicates. The remaining parameters were kept at default settings, and this analysis was performed using the online server IQTREE [29]. The resulting phylogenetic tree was edited using iTOL software v5 [30].

## 3. Results

All pool samples analyzed by nested PCR were negative for the detection of coronavirus.

A total of 106,082.436 reads were generated through metagenomic sequencing of fecal pool samples, resulting in 36,196.466 cleaned reads after quality processing. All contigs were aligned against the SARS-CoV-2 Wuhan-1 strain (MN908947.3). We identified a sequence with a minimum depth of two reads, matching a 250 bp region of the RdRp gene (maximum depth was eight reads). This contig was obtained from pool 9 collected from white-fronted capuchins (*Cebus unicolor*) at a rescue center from Bello Horizonte, Ucayali region and submitted to NCBI with the accession number: OR418346.1. Appendix A displays the alignment of the eight reads and the coverage in the contig sequence obtained during this study.

**Figure 2:**
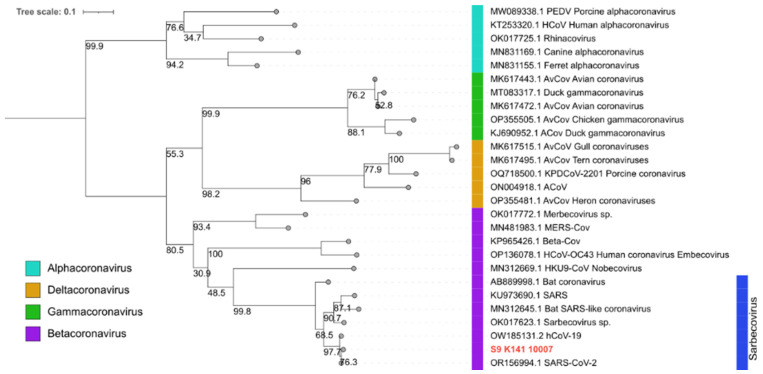
Maximum likelihood phylogenetic tree depicting 27 RdRp (RNA polymerase RNA dependent) partial nucleotide sequences from coronaviruses, classified into four genus (*Alphacoronavirus*, *Betacoronavirus*, *Gammacoronavirus* and *Deltacoronavirus*) and four subgenera: *Embecovirus* (KP965426 and OP136078), *Merbecovirus* (OK017772 and MN481983), *Nobecovirus* (MN312669), and *Sarbecovirus* (AB889998, KU973690, MN312645, OK017623, OW185131, and OR156994) of the *Betacoronavirus* genus. Blue bar represents sequences grouped within the Sarbecovirus subgenera. The sequence generated in this study is highlighted in red. Bootstrap values are displayed below and to the left of the nodes. The scale bar indicates genetic distance estimated by using the GTR + F + I + G4 substitution model implemented in IQTREE.

We aligned the partial RdRp sequence (250 bp) of the *orf1ab* gene obtained from white-fronted capuchins (*Cebus unicolor*) with homologous sequences representative of the *Sarbecovirus* subgenus and other subgenera (*Merbecovirus*, *Embecovirus*, *Nobecovirus*) within the *Betacoronavirus* genus, obtained from animal and human hosts. Additionally, sequences from other coronavirus genera (*Alphacoronavirus*, *Deltacoronavirus*, and *Gammacoronavirus*) were included, all retrieved from the GenBank database. A maximum-likelihood phylogenetic tree was constructed (Figure 2). Our sequence clustered with other SARS-CoV-2 sequences (OR156994.1 and OW185131.1) within the *Sarbecovirus* clade, confirming the detection of SARS-CoV-2 in our sample.

## 4. Discussion

Natural infections of SARS-CoV-2 in both domestic and wild animals have been documented worldwide since the onset of the COVID-19 pandemic [31]. The spillover of the virus from humans to animals can alter its pathogenicity and transmissibility within different host species. Therefore, conducting surveillance investigations in animals is imperative to monitor and prevent the emergence of potential new SARS-CoV-2 variants [15]. In this study, we report the detection of SARS-CoV-2 RNA sequences in fecal samples from captive white-fronted capuchins (*Cebus unicolor*) as part of a surveillance study that included 76 semi-captive and captive New World monkeys across three regions of the Peruvian Amazon.

SARS-CoV-2 RNA is commonly excreted in the feces of infected individuals [32]. This characteristic holds significant implications for tracking and monitoring SARS-CoV-2 variants through wastewater sequencing [33,34].

Various factors may affect the detection of viruses through molecular techniques, including low levels of virus excretion, the presence of PCR inhibitors hindering reverse transcription reactions, and the presence of RNAses in fecal matter capable of degrading RNA molecules [32,35]. These limitations could impact our ability to detect viral sequences using molecular techniques such as Nested PCR or other regions of the SARS-CoV-2 genome through metagenomic sequencing. Furthermore, there is still limited evidence regarding the relationship between clinical signs and fecal excretion of the virus in NHPs and other animals. However, in humans, it is suggested that there is no correlation between virus elimination in feces and the manifestation of gastrointestinal clinical signs or severity of disease [36].

Employing pooled samples, as conducted in this study, can also impact the sensitivity of virus detection via molecular techniques. However, it is a strategy that conserves reagents and time, and may be applicable in scenarios with low incidence rates of infections [37]. Regarding the positive pool, the low coverage and depth of the contig imply that the viral sequence likely originated from a single individual. Nonetheless, it is crucial to take into account the sample characteristics (fecal samples) and the low prevalence of this virus in New World primates [18].

The fecal samples from monkeys in the Ucayali, Loreto, and Madre de Dios regions were collected during a period of reduced SARS-CoV-2 transmission. Previously, Loreto had been severely affected by the COVID-19 pandemic and recorded the highest SARS-CoV-2 seroprevalence among the Peruvian population [38]. The Gamma variant (P.1), which emerged in Brazil in 2021, became dominant in the Peruvian Amazon, even in the presence of other variants like Lambda, suggesting a distinct transmission dynamic in the region [39]. We did not identify SARS-CoV-2 in samples from Loreto and Madre de Dios. In the case of Madre de Dios, samples were collected in February 2023 when daily infection cases in the human population were at a low level. The samples from Loreto were collected during a period of increased SARS-CoV-2 cases in Peru, between July–August 2022. However, these samples were obtained from semi-captive monkeys on two islands outside the city of Iquitos (Figure 1), where interactions with these populations were rare, thus reducing the likelihood of transmission. On the other hand, the samples from Ucayali (collected in August 2022) were taken from captive monkeys at a rescue center in Bello Horizonte where the animals had contact with caregivers. The presence of SARS-CoV-2 in captive wild animals has been reported in various zoos in the USA, with suggestions that asymptomatic zoo employees may have infected these animals [40]. A similar scenario could have occurred with the white-fronted capuchins (*Cebus unicolor*) since these animals were in contact with caregivers upon arrival from the rescue center, making it likely that the source of the infection in the monkeys originated from some of the employees.

In NHPs, there is growing concern regarding SARS-CoV-2 infection. Predictive studies involving structural and comparative analysis of viral-cell receptor Angiotensin-converting enzyme 2 (ACE2) in vertebrates, including NHPs, have indicated a broad host range for SARS-CoV-2. This categorizes Old World and New World primates into the very high and medium risk groups, respectively [41,42]. In Brazil, from 2019 to 2021, free-living neotropical NHPs from the *Atelidae*, *Callitrichidae* and *Pitheciidae* families were evaluated, with no evidence of SARS-CoV-2 infection found in NHPs residing in urban, rural, or rainforest habitats [43,44]; However, the first documented case of natural SARS-CoV-2 infection in neotropical NHPs was recently reported in a Free-Ranging Black-Tailed Marmoset (*Mico melanurus*) [18].

Our study represents the first report of SARS-CoV-2 detection in captive wild non-human primates, specifically white-fronted capuchins (*Cebus unicolor*), in Peru. These findings suggest that SARS-CoV-2 may already be infecting neotropical NHPs. However, the low coverage (2–7-fold sequencing depth) of viral sequences suggests a low virus load in the collected fecal samples, implying that individuals may have been exposed to the virus from infectious sources. Exposure to low viral concentrations and susceptibility to infection are drivers that can determine the initiation of an infectious process following exposure to a virus [45,46]. The findings of this study do not provide evidence of the susceptibility of *Cebus unicolor* to the SARS-CoV-2 virus infection; however, this underscores the critical importance of implementing measures to prevent the transmission of pathogenic agents from humans to captive wild animals or those in close proximity to humans.

The combination of a high-density population, extensive geographic exploitation, illegal wildlife trade, and inadequate sanitation oversight form a dangerous scenario that can facilitate disease outbreaks [47]. The Peruvian Amazon serves as a clear example of this perfect storm. Additionally, various studies suggest that the prevalence of infectious agents increases among captive wildlife populations [48], where interactions among different animal species can pose a potential risk for the transmission of various pathogenic agents. This includes instances of anthroponotic transmission, as suggested in our study. This risk is particularly elevated when caregivers do not provide adequate care.

Based on our results, we strongly urge individuals to take sanitation precautions when coming into contact with any species of wild mammals, including NHPs, in order to minimize the risk of SARS-CoV-2 transmission from humans to wildlife. Such transmission can have potentially devastating consequences for both humans and wild animals [49,50]. Given the findings of our study, we also recommend continuous monitoring of domestic and wild mammal populations to detect signs of SARS-CoV-2 and proactively mitigate future risks.

## 5. Conclusions

SARS-CoV-2 viral RNA was detected in fecal samples of white-fronted capuchins (*Cebus unicolor*) housed in captivity in the Ucayali region of the Peruvian Amazon. To the best of our knowledge, this marks the first documented instance of SARS-CoV-2 detection in NHPs from the Peruvian Amazon. These findings suggest that NHPs may acquire the virus from sources such as SARS-CoV-2-infected humans, emphasizing the adverse impact of anthropogenic activities on the human–NHP interface. Further research is essential to monitor animal species as potential hosts of SARS-CoV-2. Additionally, enhancing the epidemiological surveillance of this virus in wild populations and human communities is crucial for a comprehensive understanding of the prevalence, transmission dynamics, and consequences of coronaviruses in diverse wild animal populations.

## Figures and Tables

**Table 1:** List of pools of NHP stool samples organized according to species and sampling areas (N = 76).

Region	Sampling Area	NHP Species	Pooled Samples	# Pool
Madre de Dios	South of Puerto Maldonado	Red howler monkey (*Alouatta seniculus*)	7	Pool 1
Madre de Dios	Northeast of Puerto Maldonado	Black spider monkey (*Ateles chamek*)	7	Pool 2
Madre de Dios	Northeast of Puerto Maldonado	Red howler monkey (*Alouatta seniculus*)	5	Pool 3
Madre de Dios	South of Puerto Maldonado	White fronted capuchin (*Cebus unicolor*)	7	Pool 4
Ucayali	Bello Horizonte	Black spider monkey (*Ateles chamek*)	11	Pool 5
Loreto	Muyuy	White lipped tamarin (*Saguinus labiatus*)	9	Pool 6
Loreto	Padre Isla	Moustached tamarin (*Saguinus mystax*)	8	Pool 7
Madre de Dios	Northeast of Puerto Maldonado	Black spider monkey (*Ateles chamek*)	7	Pool 8
Ucayali	Bello Horizonte	White fronted capuchin (*Cebus unicolor*)	9	Pool 9
Madre de Dios	South of Puerto Maldonado	Red howler monkey (*Alouatta seniculus*)	6	Pool 10
Total samples			76	

**Figure A1:**
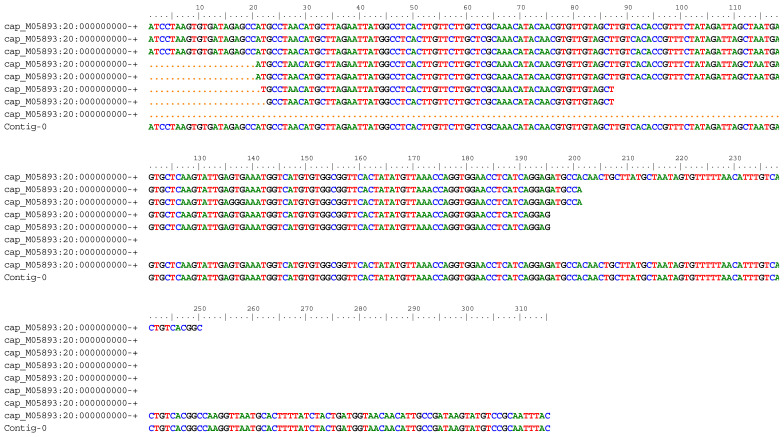
Alignment of 8 reads (250, 201, 201, 175, 175, 66, 66, and 195 nucleotides in length) from the pool 9. The alignment of reads covering the region (from position 15,296 to 15,610 of Wuhan-1 strain, GenBank accession number: MN908947) that contain the *orf1ab* or RdRp gene (position 266 to 21,544) is shown. The figure was generated using the CAP (contig assembly program) application from Bioedit software v. 7.2.5.

## Data Availability

The SRA (Sequence Read Archives) were deposited in the NCBI SRA Database under BioProject accession number PRJNA1065468. The FASTA files obtained through by New Generation sequencing were deposited in the NCBI (https://www.ncbi.nlm.nih.gov/nuccore/OR418346.1; accessed on 12 August 2023) under accession number: OR418346.1.
